# Inhibition of PGE2 in Subchondral Bone Attenuates Osteoarthritis

**DOI:** 10.3390/cells11172760

**Published:** 2022-09-05

**Authors:** Qi Sun, Yuanzhen Zhang, Yilan Ding, Wenqing Xie, Hengzhen Li, Shaohua Li, Yusheng Li, Ming Cai

**Affiliations:** 1 Department of Orthopaedics, Shanghai Tenth People’s Hospital, School of Medicine, Tongji University, Shanghai 200072, China; 2 Department of Orthopedics, Xiangya Hospital, Central South University, Changsha 410008, China; 3 National Clinical Research Center for Geriatric Disorders, Xiangya Hospital, Central South University, Changsha 410008, China

**Keywords:** subchondral bone, PGE2, osteoarthritis, bone remodeling, sensory nerve, COX2

## Abstract

Aberrant subchondral bone architecture is a crucial driver of the pathological progression of osteoarthritis, coupled with increased sensory innervation. The sensory PGE2/EP4 pathway is involved in the regulation of bone mass accrual by the induction of differentiation of mesenchymal stromal cells. This study aimed to clarify whether the sensory PGE2/EP4 pathway induces aberrant structural alteration of subchondral bone in osteoarthritis. Destabilization of the medial meniscus (DMM) using a mouse model was combined with three approaches: the treatment of celecoxib, capsaicin, and sensory nerve-specific prostaglandin E2 receptor 4 (EP4)-knockout mice. Cartilage degeneration, subchondral bone architecture, PGE2 levels, distribution of sensory nerves, the number of osteoprogenitors, and pain-related behavior in DMM mice were assessed. Serum and tissue PGE2 levels and subchondral bone architecture in a human sample were measured. Increased PGE2 is closely related to subchondral bone’s abnormal microstructure in humans and mice. Elevated PGE2 concentration in subchondral bone that is mainly derived from osteoblasts occurs in early-stage osteoarthritis, preceding articular cartilage degeneration in mice. The decreased PGE2 levels by the celecoxib or sensory denervation by capsaicin attenuate the aberrant alteration of subchondral bone architecture, joint degeneration, and pain. Selective EP4 receptor knockout of the sensory nerve attenuates the aberrant formation of subchondral bone and facilitates the prevention of cartilage degeneration in DMM mice. Excessive PGE2 in subchondral bone caused a pathological alteration to subchondral bone in osteoarthritis and maintaining the physiological level of PGE2 could potentially be used as an osteoarthritis treatment.

## 1. Introduction

Osteoarthritis is the most common chronic joint disorder globally and it affects approximately 18% of women and 10% of men over 60 years old [1]. It is one of the leading causes of disability and pain and carries a tremendous socioeconomic cost [1,2]. Although joint arthroplasty is effective for symptomatic end-stage osteoarthritis, it has several disadvantages, including poor functional outcomes and limited lifespan of prostheses [3,4]. Therefore, the current focus must shift to the prevention and treatment of early-stage osteoarthritis. However, available osteoarthritis treatments are not currently disease-modifying [1,5,6], meaning that the morbidity associated with early-stage osteoarthritis cannot be sufficiently addressed due to a limited understanding of the pathogenesis. The advanced degeneration of articular cartilage is the primary concern of osteoarthritis and it is regarded as the critical driving cause of the disorder [7]. However, targeting the pathways of articular cartilage degeneration may be insufficient for the attenuation of osteoarthritis progression [7,8].

Recent evidence has indicated that aberrant subchondral bone plays a causal role in the development of osteoarthritis [9,10,11,12,13,14]. Current concepts focus on the functional unit that is formed by articular cartilage and subchondral bone [15,16,17]. Subchondral bone is continuously challenged and undergoes biomechanical adaptions, receiving and dissipating the mechanical stress that is transduced from the overlying articular cartilage and providing mechanical support during loading and movement [15,16,17]. Structural alterations to subchondral bone have been reported to precede the degeneration of articular cartilage in human osteoarthritis [18,19]. High-level remodeling of subchondral bone has also been observed in osteoarthritis, particularly in the early stage, which consequently leads to the microenvironment of subchondral bone being altered [10]. Aberrant subchondral bone formation may result in subchondral bone sclerosis and an increased thickness of the subchondral plate, ultimately accelerating articular cartilage degeneration [9,10,11,20,21]. However, the mechanism of aberrant subchondral bone formation during the development of osteoarthritis remains unclear.

Sensory innervation in the bone tissue is involved in the regulation of bone mass accrual and the promotion of bone regeneration [22,23,24,25]. Sensory nerve denervation caused by capsaicin or genetic mouse models (Advillin-Cre: TrkA *^flox/flox^*) significantly inhibits osteogenesis in mice [23]. An increased density of sensory nerves in subchondral bone is linked to joint mechanical hyperalgesia and pathological subchondral bone formation in osteoarthritis [9,17,26,27]. Prostaglandin E2 (PGE2) is a product of arachidonic acid processed by cyclooxygenase 2 (COX-2) that activates sensory nerves as an inflammatory mediator. It causes joint pain hypersensitivity and sensory sensitization by primarily binding to the PGE2 receptor 4 (EP4) of sensory neurons [9,25,26,28]. Previous literature has demonstrated that PGE2 activates the EP4 of the sensory neuron, inducing mesenchymal stromal cells (MSCs) into osteoblasts [22,23]. PGE2 levels in subchondral bone have been reported to increase with subchondral bone alteration during osteoarthritis, which stimulates osteoarthritis pain by mainly binding to the EP4 of the sensory nerve [9,26].

Recently, the role of subchondral bone PGE2 in osteoarthritis progression has been investigated [29,30,31]. Elevated PGE2 levels have been found to cause aberrant subchondral bone in spontaneous osteoarthritis and rheumatoid arthritis [29]. Increased PGE2/EP4 signaling promotes osteoarthritis progression, which is mediated by osteoclast-induced angiogenesis [30]. Subchondral bone remodeling is also controlled by sensory innervation [25], but whether the regulation of sensory nerves is affected by PGE2 in subchondral bone formation remains unclear. In this study, the role and mechanism of PGE2 in the aberrant formation of subchondral bone during osteoarthritis progression was examined. It was found that PGE2 signaling increased sensory innervation in the subchondral bone starting at the early stage in the DMM osteoarthritis model, which results in increased and uneven subchondral bone formation and joint mechanical hyperalgesia. The inhibition of sensory PGE2 signaling is sufficient for the attenuation of aberrant subchondral bone remodeling, articular cartilage degeneration, and osteoarthritis pain.

## 2. Methods and Materials

### 2.1. Mice Preparation and Treatment

We purchased adult male C57BL/6J (wild type, WT) mice from Shanghai Model Organisms. The Advillin-Cre mouse strain (stock no.: 032536) and the EP4 *^flox/flox^* mouse strain (stock no.: 028102) were purchased from Jackson Laboratory. Heterozygous Advillin-Cre mice (male) were crossed with EP4 *^flox/flox^* mice (female). We intercrossed the offspring, then obtained the following genotypes: WT, Advillin-Cre, and Advillin-Cre:EP4 *^flox/flox^* (herein as EP4^−/−^). Advillin-Cre forward, AATGGCTCCCTGTTCACTGT, reverse, AGGCAAATT TTGGTGTACGG; WT: TGACTAGGTAGAGGTGCAAATGTC; EP4 loxP allele forward, GGCGGGATCAGTTAGATGG, reverse, GTGAAGCGAGTCCTTAGGC. We anesthetized experimental mice with a mixture of ketamine and xylazine (ketamine, 80 mg/kg; xylazine, 4 mg/kg) and then performed sham or DMM surgery on the left knee. For the time-course experiment, the samples were harvested after animals’ euthanasia at 0, 1, 2, 4, 6, and 8 weeks after DMM or sham surgery (n = 5–8). We applied either different doses (1, 5, 25, 125 mg/kg) of celecoxib (Sigma-Aldrich, PZ0008) or the equivalent volume of phosphate-buffered saline (PBS) via oral administration daily for 8 weeks immediately after surgery. We injected capsaicin (Millipore Sigma, M2028, 30 mg/kg) or the equivalent volume of PBS via intraperitoneal administration daily for 4 weeks. A total of 406 mice were used in this study (detailed mice allocation and mice number can be seen in Table S1).

### 2.2. DMM Osteoarthritis Model

We performed DMM surgery on the left knee joints of three-month-old male C57BL/6 mice, as described by previous research [32]. Briefly, adult mice were randomly attributed to either sham or DMM groups. These mice were anesthetized by a mixture of ketamine and xylazine. We transected the anterior medial meniscotibial ligament of the knee joint with microscissors in the DMM group. The anterior medial meniscotibial ligament was just exposed but not transected in the sham group.

### 2.3. Immunocytochemistry, Immunofluorescence, and Histomorphometry

The mice’s knee joints were stored in 70% ethanol at 4 °C after being fixed in 4% paraformaldehyde overnight. These specimens were decalcified in 10% ethylenediaminetetraacetic acid (EDTA, pH 7.35–7.45) for 14 days and were embedded in paraffin or Optimal Cutting Temperature (Sakura Finetek, Torrance, CA, USA) Compound. The section of the medial compartment of the knee joint was cut in a sagittal-oriented fashion. Then, 30 μm thick sections were used to detect sensory nerves via immunofluorescent staining. Sections of 4 μm thickness were used for TRAP staining (Sigma-Aldrich, 387A-1KT), Safranin O/fast green (SOFG) staining, and immunohistochemistry staining following a standard protocol. Briefly, the sections of the knee joint were incubated overnight with primary antibodies at 4 °C in a humidifier chamber (Table 1). For immunohistochemical staining, we used a horseradish peroxidase streptavidin detection system (Dako, Agilent Technologies) to retrieve the immune activity, counterstained by hematoxylin (Sigma-Aldrich). For immunofluorescence staining, corresponding secondary antibodies were used to detect the immune activity (at room temperature for 1 hour, avoiding light) (Table 1). Subsequently, 4′,6-diamidino-2-phenylindole (DAPI, Invitrogen, Carlsbad, CA, USA) was used for counterstaining. An Olympus BX53 microscope and Zeiss LSM 880 confocal microscope were used to capture sample images. Image J software (Version 1.49, Media Cybernetics Inc, Rockville, MD, USA) was used for quantitative histomorphometric analysis. We calculated the OARSI scores of the SOFG staining, as previously described [33]. 

### 2.4. μCT Analysis

The mice’s knee joints were scanned by a high-resolution Micro CT (Skyscan 1172, Bruker). In detail, the parameters were set as follows: voltage, 50 kV; current, 200 μA; resolution, 9.0 μm per pixel. The joint images were processed and analyzed with NRecon software v1.6 and CTAn software v1.9. The images of the reconstructed subchondral bone were processed by the software CTVol v2.0 for 3-dimensional visualization. The region of interest is defined as the whole medial plateau of tibial subchondral bone. Nine consecutive images of the medial compartment of subchondral bone were used for 3D reconstruction. The following 3-dimensional structural parameters of subchondral bone were involved: bone fraction (BV/TV): bone volume/tissue volume, Tb.Sp: trabecular bone separation, and SBP Th: subchondral bone plate thickness. 

### 2.5. ELISA Quantification of PGE2

The concentration of PGE2 in serum, subchondral bone, or cartilage extracts was measured using a PGE2 human ELISA kit (KHL1701, ThermoFisher Scientific) and PGE2 assay kit (R&D Systems, Bio-Techne), according to the manufacturer’s instructions. To prepare cartilage or subchondral bone extracts of mice, tibial subchondral bones and articular cartilage were isolated and dissected from soft tissues and connective tissues on ice. Tibial subchondral bone was carefully dissected from articular cartilage on ice under a microscope. Liquid nitrogen was used to flash-freeze these specimens, which were subsequently pulverized in frozen pulverizers. Then, we transferred this tissue powder to pre-frozen Eppendorf tubes with RIPA buffer and placed them on ice for 30 minutes. The sample was collected and stored at −80 °C after 1 hour of rotation at 4 °C (cold room). After centrifuging at 2000 rpm for 10 min, the serum was collected for analysis or stored at −80 °C.

### 2.6. Human Sample

We collected serum from eight individuals with knee osteoarthritis who underwent total knee arthroplasty pre-operatively and eight young adults with normal joints. Both the young adults and osteoarthritis patients had not taken non-steroidal anti-inflammatory drugs (NSAIDs) for a minimum of three months. The demographic data of patients were collected (Table 2). All tibial plateau specimens were collected from those osteoarthritis patients who underwent total knee arthroplasty (the detailed individual patient Kellgren–Lawrence (KL) scores are listed as followed: KL1 -1, KL-2, KL-2, KL-2, KL-3, KL-4, KL-4, KL-4). As described in Table 3, cartilage with underlying subchondral bone was classified and separated into three groups: non-lesion, moderate lesion, and lesion area. Briefly, upon collection after TKA, the tibial plateau was separated using an oscillating saw, according to the cartilage degeneration grade. Then, the PGE2 extraction process was the same as the “ELISA quantification of PGE2”.

### 2.7. HR-QCT Analysis

The microstructure of the human knee subchondral bone was assessed by high-resolution peripheral quantitative computed tomography (HR-QCT) (XtremeCT I, Scanco Medical, Brüttisellen, Switzerland) 1 week before total knee arthroplasty. The parameters were set as follows: voltage: 120 kV, current: 260 mA, and resolution: 400 μm per pixel. The images were exported in ISQ file format, and were subsequently processed and analyzed with NRecon software v1.6 and CTAn software v1.9, respectively. The images of the reconstructed subchondral bone were processed by the software CTVol v2.0 for 3-dimensional visualization. The subchondral bone structural parameters, including BV/TV, Tb.Sp, and SBP Th, were analyzed. 

### 2.8. Von Frey Test

Allodynia was determined using von Frey hairs in ascending order (bending force range ≈ 0.07, 0.40, 0.60, 1.0, 1.4, 2.0, 4.0, or 6.0 g). These von Frey hairs were held perpendicular to the left hind paw (the plantar surface) for 2–3 s. If unresponsive, the hair in the next bend force was used until the maximum bending force level (6.0 g) was reached. In the case of a withdrawal response, the hair in the next descending order was used until there was no response. Once the first difference was observed, four more tests were performed. The 50% paw withdrawal threshold was calculated by the following formula:

50% paw withdrawal threshold = 10^(kδ+Xf)^/10,000 (1)

where k is the value for the pattern of the last six positive/negative responses, δ is the mean difference between bending force in log units, and Xf is the value of the final hair used in log units. The measurement was repeated twice at an interval of 30 min. The average value determines the 50% pressure withdrawal threshold. 

### 2.9. Pressure Threshold

The pressure hyperalgesia was measured by withdrawal thresholds of the left knee. Briefly, we applied direct and evenly increasing pressure on the knee joint, specifically the medial part. The threshold was determined by a sensor tip (diameter: 5 mm) of a force gauge (SMALGO algometer; Bioseb, Pinellas Park, FL, USA). Restrained gently by the investigator, the pressure force uniformly increased at 50 g/s until the mice could not bear the pressure, then the value was recorded. A force of 500 g was set as a cutoff to prevent additional damage to the knee joint. We used BIO-CIS software (Bioseb) to record the curve of the pressure force to ensure the uniformed increase of force. The measurement was repeated twice at an interval of 15 min. The final threshold was defined as the mean value of the two tests.

### 2.10. Voluntary Wheel Running Measurement

Mice were placed in mouse cages furnished with a running wheel with a concave surface inside. Mice were housed individually and allowed to run freely. The system equipment (Campden Instruments, Loughborough, UK) was used for running data capture that comprises signals transmitted electronically from the rotating wheels. Measurements were recorded for 24 hours. The parameters including active time, distance traveled, and mean speed were recorded and calculated. 

### 2.11. Double Labeling and Histomorphometry Analysis

We performed double labeling to evaluate the status of dynamic bone remodeling by calcein. Briefly, 0.1% calcein (10 mg/kg, Sigma-Aldrich, C0875; dissolved in PBS) was injected into the mice intraperitoneally at nine days and two days before sacrifice. Mice knee joints were stored in 70% ethanol at room temperature for two days and sectioned without decalcification. Fluorescence microscopes were used to capture labeling images for further analysis.

### 2.12. Statistics

All data were summarized and are shown as mean ± standard deviation. For comparisons between two groups, we used unpaired 2-tailed Student’s *t*-tests. For comparison among multiple groups, a 2-way ANOVA followed by the Turkey’s post hoc test was used. The differences in PGE2 and subchondral bone parameters between OA and the normal group were re-analyzed with a multivariate analysis of variance followed by LSD analysis by adjusting age, sex, and BMI. For all experiments, *p* < 0.05 was regarded as statistically significant (* *p* < 0.05, ** *p* < 0.01, and *** *p* < 0.001). The sample size was not predetermined by the statistical method. All data analyses were performed using SPSS 19.0 software (IBM). According to pre-established inclusion and exclusion criteria, samples or animals were not excluded. The experiments were performed randomly, and the investigators responsible for experiments and outcomes were blinded to allocation. 

### 2.13. Study Approval

Written informed consent of all involved patients and approval of the institutional investigational review board of Shanghai Tenth People’s Hospital, Tongji University was obtained prior to image capture and harvesting of human tissue samples. All work described above was carried out according to the code of ethics of The World Medical Association (Declaration of Helsinki). Involved animals were maintained in the animal facility of Tongji University. The full experimental animal protocol was reviewed and approved by the institutional animal care and use committee of Shanghai Tenth People’s Hospital, Tongji University.

## 3. Results

### 3.1. Elevated PGE2 in Subchondral Bone Occurs during an Early Stage in DMM Mice

**Figure 1:**
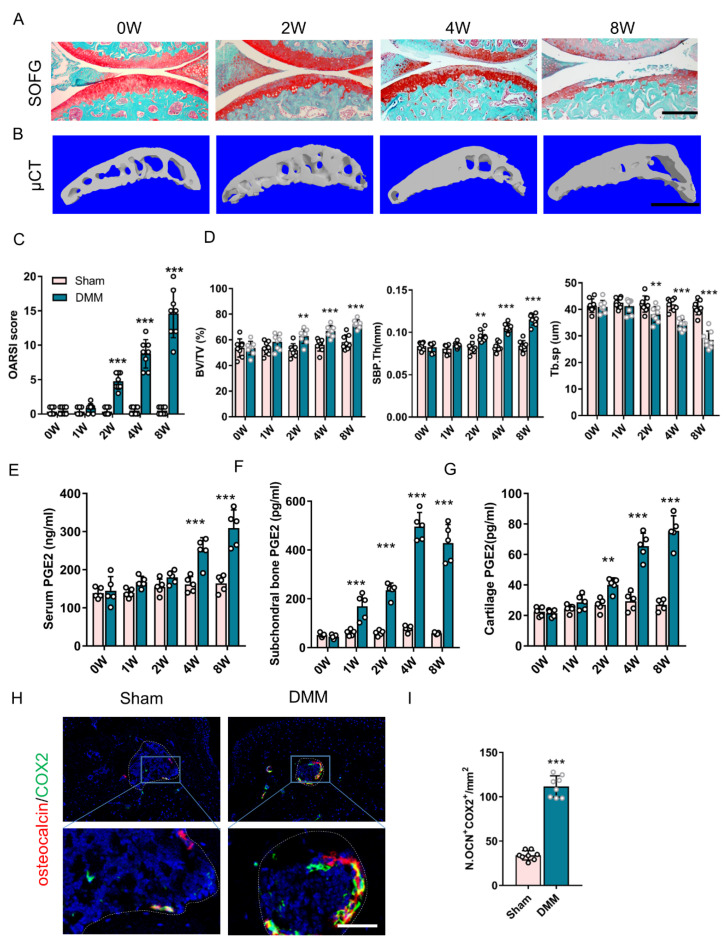
PGE2 level is highly related to joint degeneration in the DMM model. (**A**) Safranin O/Fast green (SOFG) staining of the medial compartment of mouse tibial subchondral bone (sagittal view) at zero, two, four, and eight weeks following DMM surgery. Scale bar: 200 μm. (**B**) Three-dimensional microcomputed tomography (μCT) images of the medial compartment of the tibial subchondral bone (sagittal view). Scale bar: 500 μm. (**C**) Osteoarthritis Research Society International (OARSI) scores. n = 8 mice per group. (**D**) Quantitative analysis of the structural parameters of subchondral bone: bone fraction (BV/TV), trabecular bone separation (Tb.sp), and subchondral bone plate thickness (SBP.Th). n = 8 mice per group. (**E**–**G**) ELISA analysis of PGE2 concentrations in the serum (**E**), subchondral bone (**F**), and articular cartilage (**G**) of mice following DMM or sham surgery. n = 5 mice per group. (**H**,**I**) Representative double immunofluorescent images (**H**) and quantitative analysis (**I**) of osteocalcin (red) and COX-2 (green) in subchondral bone from DMM or sham mice 4 weeks after surgery. Scale bar: 30 μm. n = 8 mice per group. ** *p* < 0.01 and *** *p* < 0.001, means non-significant.

To examine alterations in the PGE2 level in osteoarthritis development, posttraumatic osteoarthritis was induced by establishing the DMM model. A mild loss of proteoglycan in the articular cartilage was first detected two weeks after DMM surgery and was aggravated at eight weeks (Figure 1A). It is notable that there was a rough surface of the articular cartilage at four weeks and an extensive lesion of the tibial articular cartilage at eight weeks following DMM surgery (Figure 1A). Similarly, the Osteoarthritis Research Society International (OARSI) score demonstrated that articular cartilage degeneration began at two weeks, becoming progressively severe at eight weeks following surgery (Figure 1C). Three-dimensional μCT analysis showed that the subchondral bone fraction (bone volume/tissue volume, BV/TV) increased from two weeks after surgery (Figure 1B,D), which suggests the formation of abnormal subchondral bone in osteoarthritis. Increased thickness in the subchondral bone plate (SBP.Th) and decreased trabecular bone separation (Tb.Sp) in DMM mice lasted from two to eight weeks following DMM surgery (Figure 1B,D), which indicates uneven bone formation in osteoarthritis. 

Osteoarthritis pain was assessed using pain-related behavior tests, including 50% pressure withdrawal threshold, pressure threshold, and voluntary wheel running measurements. Moreover, the 50% pressure withdrawal threshold exhibited a significant decrease from two to eight weeks following DMM surgery, which indicates hyperalgesia of the knee joints as a response to mechanical stimuli in osteoarthritis (Figure S1A). It was found that the pressure threshold decreased significantly at four and eight weeks following DMM surgery (Figure S1B). Spontaneous activity, such as distance traveled, mean speed, and active time per 24 h, decreased significantly at four and eight weeks following DMM surgery in comparison to the sham controls (Figure S1C–E). These results suggest the DMM-induced development of hyperalgesia.

The PGE2 levels were then examined, which were related to bone homeostasis and osteoarthritis pain [22,23,26]. A significantly increased concentration of serum PGE2 was found at four weeks and was sustained to eight weeks following DMM surgery (Figure 1E). The PGE2 levels in subchondral bone and articular cartilage were measured separately. An increased PGE2 level of subchondral bone was initiated one week following DMM surgery, before cartilage at two weeks (Figure 1F,G). Subchondral bone PGE2 levels were up to eight times greater than those of the sham controls, compared to a triple increase in articular cartilage. The COX-2 expression showed a global increase in the DMM joint (Figure S2), which specifically exhibited an approximately fourfold increase and was primarily expressed in the osteocalcin-positive osteoblastic cells following DMM surgery when compared to the sham controls (Figure 1H,J). These results suggest that the uneven formation of subchondral bone and hyperalgesia are related to elevated levels of PGE2 in subchondral bone in osteoarthritis. 

### 3.2. Elevated PGE2 Is Closely Associated with the Microstructural Alteration of Subchondral Bone in Human Osteoarthritis

**Figure 2:**
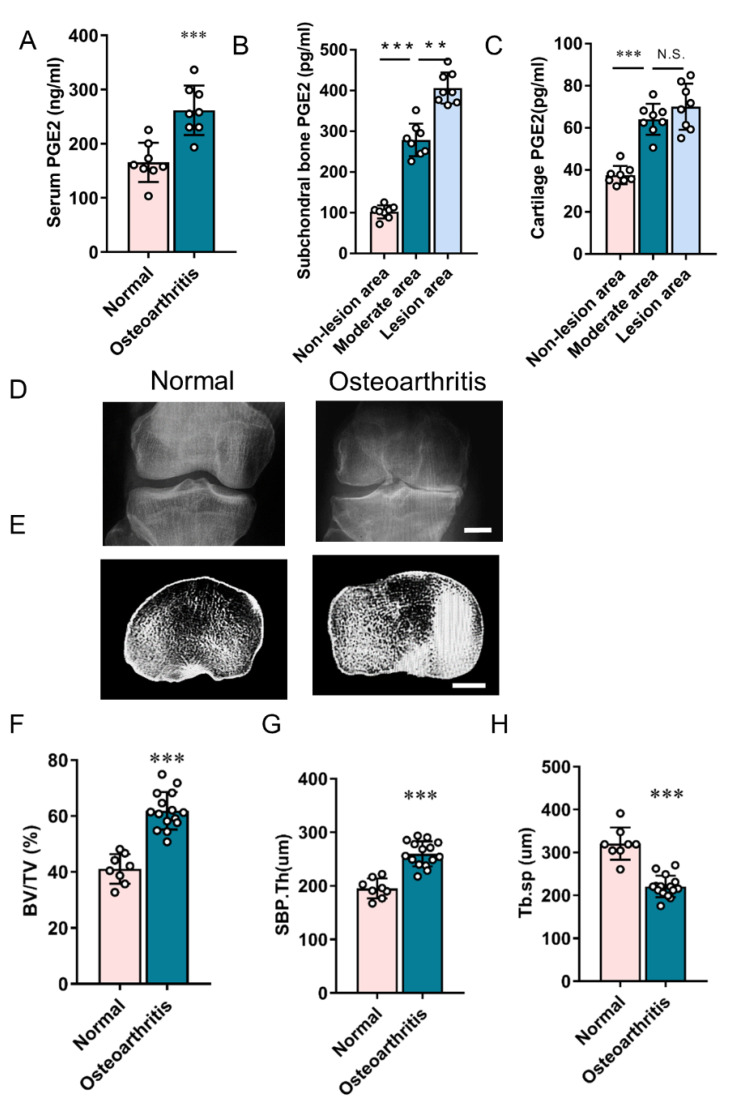
Increased PGE2 levels and aberrant subchondral bone architecture occur in human osteoarthritis. (**A**–**C**) ELISA quantitative analysis of PGE2 concentration in the serum (**A**), subchondral bone (**B**), and articular cartilage (**C**) in humans. (**D**) X-ray images of the knee joint (anteroposterior view) in normal and osteoarthritis humans. Scale bar: 20 mm. (**E**) Representative HR-QCT images (horizontal view) of tibial subchondral bone in normal and osteoarthritis humans. Scale bar: 20 mm. (**F**–**H**) Quantitative analysis of subchondral bone structure parameters: BV/TV (**F**), Tb.Sp (**G**), and SBP.Th (**H**). ** *p* < 0.01, *** *p* < 0.001, and N.S. means non-significant.

To examine PGE2 changes in human osteoarthritis, PGE2 concentrations in the serum were measured in normal and osteoarthritis patients. The PGE2 concentration of subchondral bone and articular cartilage was also measured in the normal area and osteoarthritic area of osteoarthritis patients who underwent total knee replacement. From ELISA analysis, the PGE2 concentration in the serum was found to be approximately 50% higher in osteoarthritis patients than in the normal controls (Figure 2A). The subchondral bone PGE2 level in the osteoarthritic area intensively increased when compared to the non-lesion area, where the PGE2 level in the lesion area was higher than in the moderate area (Figure 2B). Similarly, the cartilage PGE2 level in the osteoarthritic area intensively increased when compared to the non-lesion areas, but no significant difference was observed between moderate and lesion-area cartilage (Figure 2C). These data suggest the existence of a positive relationship between PGE2 concentration and osteoarthritis severity, specifically in subchondral bone.

Subchondral bone volume and the architectural alteration of knee joints were also explored by HR-QCT. Subchondral bone alteration is positively correlated to osteoarthritis severity (Figure 2D,E). Consistent with the DMM mice, BV/TV increased significantly in osteoarthritis patients (Figure 2F), which suggests that aberrant subchondral bone formation is related to the occurrence of osteoarthritis. The altered structural parameters of subchondral bone, including increased SBP.Th and decreased Tb.Sp, show a significant difference in osteoarthritis patients when compared to normal patients (Figure 2G,H). Therefore, these results further confirm that increased PGE2 concentration, specifically subchondral bone PGE2 concentration, is coupled with abnormal subchondral bone architecture in the progression of osteoarthritis in humans. 

### 3.3. Inhibition of PGE2 Production Attenuates Cartilage Degeneration and Pain

The effect of inhibiting PGE2 production on cartilage degeneration and pain in DMM mice was then examined. Celecoxib, a COX-2 inhibitor, was orally administered to inhibit PGE2 production. Different doses of celecoxib were given to DMM mice as a means of identifying the optimal dose (Figure S3A–E). A low dose of celecoxib had minimal effect on osteoarthritis pain relief and the protection of joint degeneration, but a higher dose, starting at 5 mg/kg/day, gradually improved osteoarthritis pain (Figure S3A–E). In addition, celecoxib was found to have an increasingly protective effect on joint degeneration with doses from 1 to 25 mg/kg. However, a more severe proteoglycan loss in articular cartilage was observed in mice that were treated with celecoxib at 125 mg/kg (Figure S3A,D). In addition, the effect of celecoxib (25 mg/kg) on the inhibition of PGE2 production in joint tissue was determined. PGE2 levels in both the subchondral bone and cartilage following DMM surgery were almost normalized through celecoxib treatment (Figure S4A,B). 

**Figure 3:**
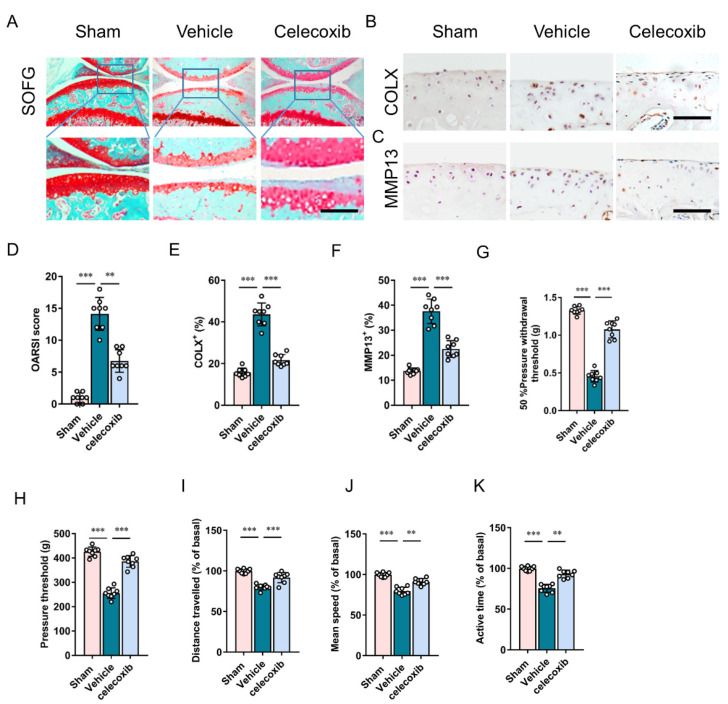
Inhibition of PGE2 prevents joint osteoarthritis degeneration and pain. (**A**) SOFG staining of the medial compartment of subchondral bone (sagittal view) at eight weeks following surgery. Scale bar: 50 μm. (**B**,**C**) Immunostaining of ColX+ (**B**) and MMP13+ (**C**) chondrocytes in articular cartilage at four weeks following surgery. Scale bar: 25 μm. (**D**) OARSI scores following surgery. n = 8 mice per group. (**E**,**F**) Quantitative analysis of ColX+ (**E**) and MMP13+ (**F**) chondrocytes. n = 8 mice per group. (**G**) Measurement of 50% pressure withdrawal threshold at eight weeks following surgery; n = 8 per group. (**H**) Measurement of the pressure threshold at eight weeks following surgery; n = 8 mice per group. (**I**–**K**) Parameters of voluntary wheel running at eight weeks following surgery: active time (**I**), mean speed (**J**), and distance traveled (**K**); n = 8 mice per group. ** *p* < 0.01, *** *p* < 0.001.

SOFG staining and the OARSI score demonstrated that celecoxib significantly attenuated articular cartilage degeneration in DMM mice (Figure 3A,D). Following immunostaining by type X collagen (COL X+) and matrix metalloproteinase 13 (MMP13+), the effect of celecoxib on the chondrocyte degeneration of articular cartilage was assessed (Figure 3B,C). Percentages of COL X+ and MMP13+ chondrocytes in DMM mice were more than twice those in the sham controls, and celecoxib dramatically reduced the magnitude of increase (Figure 3E,F), which indicates that inhibiting the increase in PGE2 attenuates articular cartilage and chondrocyte degeneration in osteoarthritis. 

Furthermore, mechanical hyperalgesia of the hind paw following DMM surgery was significantly prevented by celecoxib, as proven by an improvement of 50% pressure withdrawal threshold and pressure threshold in the celecoxib-treated DMM group in comparison to the vehicle group (Figure 3G,H). Spontaneous activities, such as distance traveled, mean speed, and active time, were reduced following DMM surgery. These decreases were almost abrogated in celecoxib-treated DMM mice (Figure 3I–K). The results indicate that inhibiting excessive PGE2 production reduces osteoarthritis pain. 

### 3.4. Inhibition of Excessive PGE2 Production Attenuates the Altered Architecture of Subchondral Bone 

**Figure 4:**
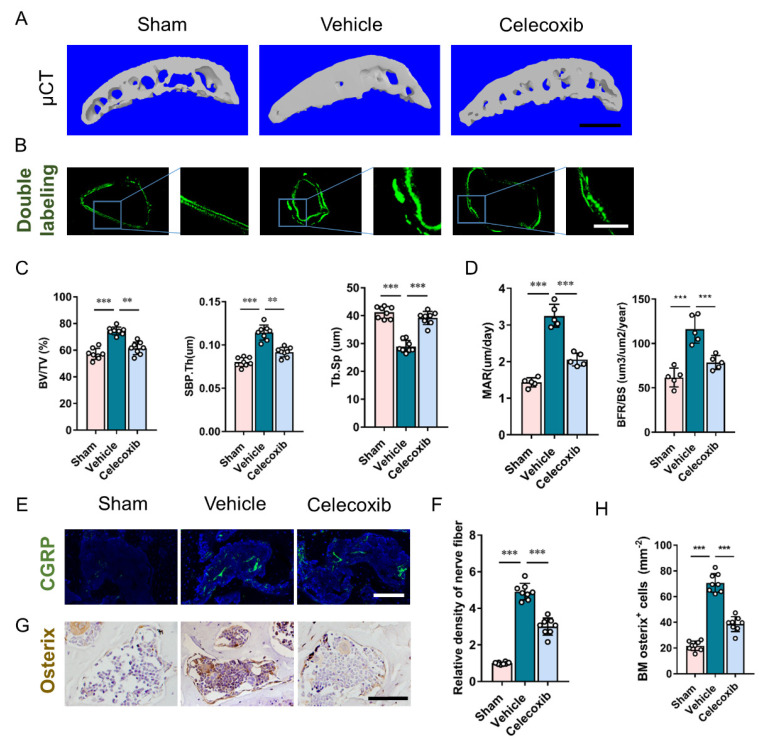
Inhibition of COX-2 attenuates uneven subchondral bone remodeling. (**A**) Three-dimensional microcomputed tomography (μCT) images of the medial compartment of tibial subchondral bone (sagittal view). Scale bar: 500 μm. (**B**) Representative images of subchondral bone with calcein double labeling at four weeks following surgery. Scale bar: 20 μm. (**C**) Quantitative analysis of subchondral bone fraction (BV/TV), Tb.Sp, and SBP.Th. n = 8 mice per group. (**D**) Quantification of calcein double labeling: mineral apposition rate (MAR) and bone formation rate (BRF). n = 5 mice per group. (**E**,**F**) Representative images (**E**) and quantitative analysis (**F**) of immunofluorescent staining of CGRP (green) + sensory nerves in subchondral bone at four weeks following surgery. Scale bar: 50 μm. (**G**,**H**) Representative images (**G**) and quantitative analysis (**H**) of immunochemical staining of osterix +(brown) osteoprogenitors in subchondral bone at four weeks following surgery. Scale bar: 50 μm. ** *p* < 0.01, *** *p* < 0.001.

Aberrant subchondral bone remodeling has been proven to induce osteoarthritis pain and joint degeneration in osteoarthritis [10]. To validate the effect and cellular mechanism of PGE2 on subchondral bone, the effect of COX-2 inhibitor on the remodeling status of subchondral bone was explored. Celecoxib prevented aberrant subchondral bone formation, indicated by a decrease in BV/TV in celecoxib-treated DMM mice (Figure 4A,C). Calcein double labeling demonstrated dramatically increased mineral apposition rate and bone formation rate in vehicle-treated DMM mice, which indicates an increased remodeling level and the formation of subchondral bone in DMM subchondral bone. These effects were abrogated by celecoxib treatment (Figure 4B,D). Improvement to the subchondral bone microarchitecture following DMM surgery with celecoxib was demonstrated by the maintenance of SBP.Th, and volume increase in Tb.Sp (Figure 4A,C). 

Sensory innervation is involved in bone homeostasis by mediating the PGE2 regulation of osteoblastic differentiation [22,23]. Sensory innervations in subchondral bone were evaluated. The calcitonin gene-related peptide (CGRP)+ sensory nerve in the subchondral bone demonstrated a five-fold increase in the vehicle-treated DMM group and a slight decrease in the celecoxib-treated DMM group (Figure 4E,F). An approximate threefold increase in osterix+ osteoblastic progenitors in subchondral bone marrow was observed in vehicle groups. The increased magnitude of osterix+ osteoblastic progenitors was inhibited by more than 50% by celecoxib in DMM mice (Figure 4G,H). 

### 3.5. Sensory Denervation Improves Subchondral Bone Architecture and Attenuates Osteoarthritis Progression

**Figure 5:**
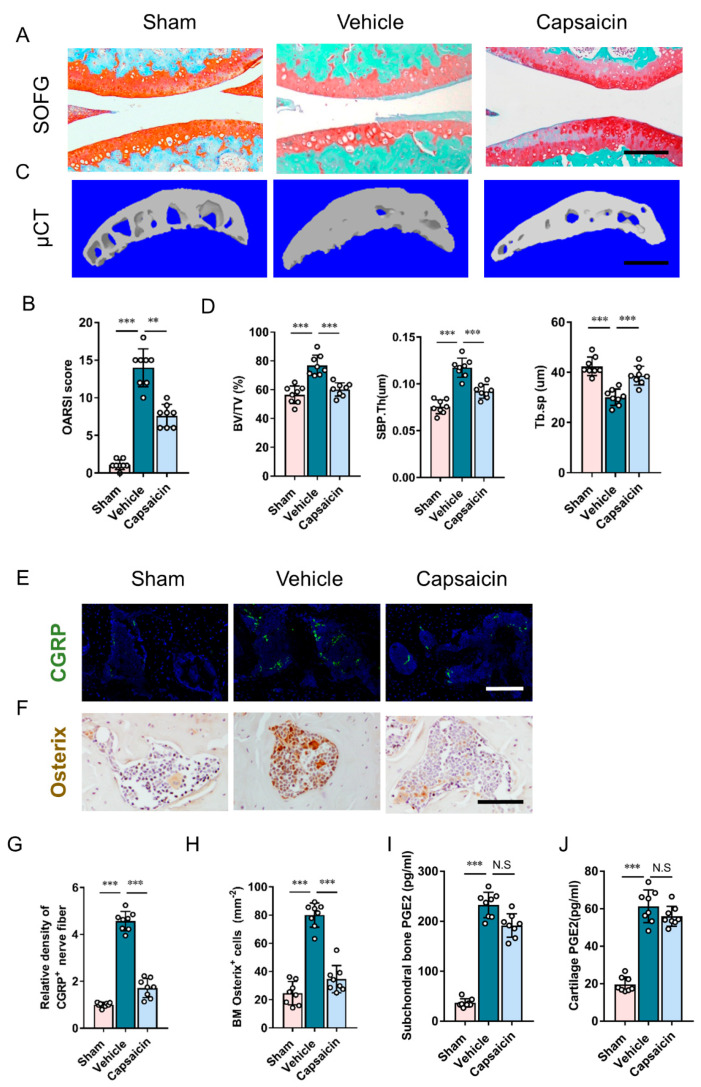
Sensory denervation stabilizes subchondral bone architecture and reduces cartilage degeneration following DMM surgery. (**A**) SOFG staining of tibia subchondral bone (sagittal section). Scale bar: 250 μm. (**B**) μCT images of subchondral bone (sagittal view). Scale bar: 500 μm. (**C**) OARSI scores at eight weeks following surgery. n = 8 mice per group. (**D**) Quantitative analysis of subchondral bone fraction (BV/TV), Tb.Sp and SBP.Th eight weeks following surgery. n = 8 mice per group. (**E**,**G**) Representative images (**E**) and quantitative analysis (**G**) of immunofluorescent staining of CGRP (green) + sensory nerves in subchondral bone at four weeks following surgery. Scale bar: 50 μm. (**F**,**H**) Representative images (**F**) and quantitative analysis (**H**) of immunochemical staining of osterix+ (brown) osteoprogenitors in subchondral bone four weeks following surgery. Scale bar: 50 μm. ELISA analysis of PGE2 concentration in tibial subchondral bone (**I**) and articular cartilage (**J**). n = 5 mice per group. ** *p* < 0.01, *** *p* < 0.001, and N.S. means non-significant.

To examine whether sensory nerves mediated PGE2 effects on subchondral bone remodeling, capsaicin was injected to eliminate sensory innervation systemically. As validated by previous research [23], the efficacy of capsaicin-induced sensory denervation is comparable to genetic mice in that TrkA was specifically knocked out from Advillin-positive sensory neurons. The proteoglycan loss of articular cartilage was attenuated by capsaicin following DMM surgery, which is consistent with the OARSI score (Figure 5A,B). Similar to celecoxib application, the microarchitecture of subchondral bone following DMM surgery was improved with the systemic application of capsaicin, as demonstrated by μCT analysis (Figure 5C,D). 

Subchondral bone sensory nerve density was significantly inhibited in the capsaicin-treated DMM mice, despite a higher density of sensory nerves in the vehicle-treated DMM mice (Figure 5E,G). In addition, the immunostaining of osterix demonstrated that capsaicin dramatically reduced osterix+ osteoblast progenitors in the subchondral bone of DMM mice (Figure 5F,H). The concentration of PGE2 in both subchondral bone and cartilage of capsaicin-treated DMM mice only exhibited a slight decrease when compared to vehicle-treated DMM mice (Figure 5I,J). In addition, capsaicin effectively attenuated the DMM-induced decrease in the 50% pressure withdrawal threshold, pressure threshold, and parameters of spontaneous activity (Figure S5A–E). Therefore, these findings suggest that sensory denervation mediates the effect of excessive PGE2 on abnormal subchondral bone remodeling and osteoarthritis pain.

### 3.6. Knockout of EP4 on Sensory Nerve Reduces Osteoarthritis Severity

**Figure 6:**
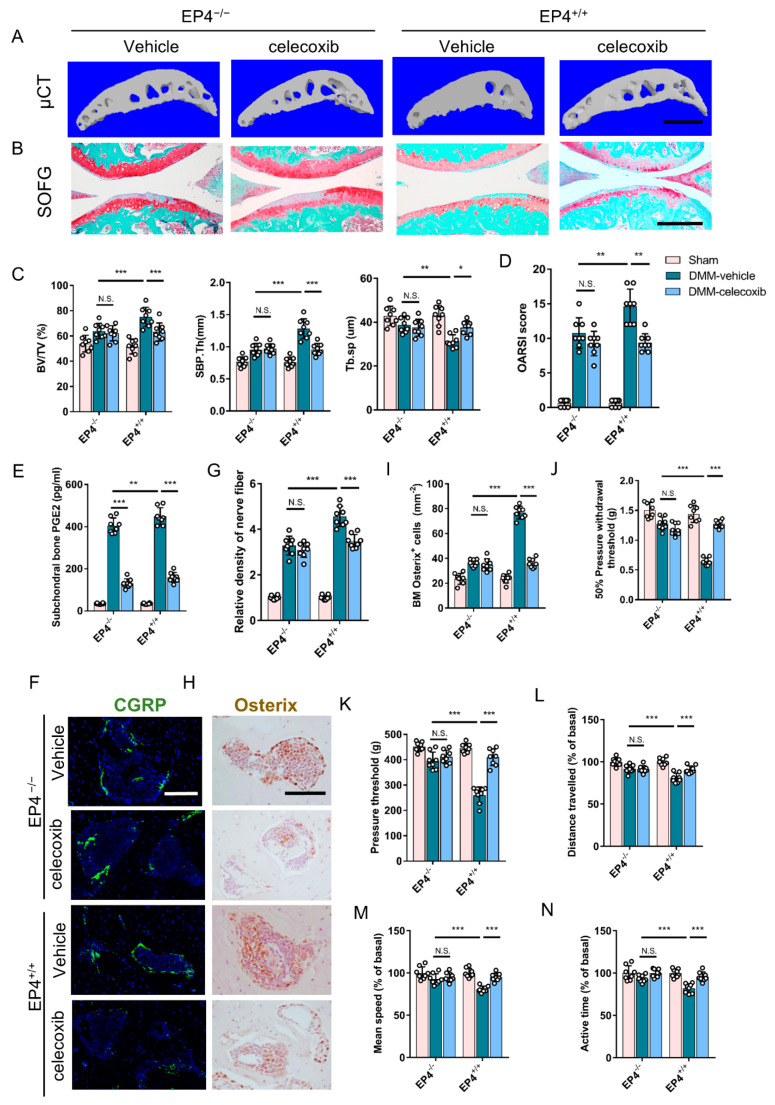
Conditional EP4 knockout mice are protected from aberrant subchondral bone architecture and osteoarthritis hypersensitivity. (**A**) μCT images of subchondral bone (sagittal view) in EP4^+/+^ mice and EP4^−/−^ (Advillin-cre-EP4) mice. Scale bar: 500 μm. (**B**) SOFG staining of tibial subchondral bone (sagittal section). Scale bar: 200 μm. (**C**) Quantitative analysis of subchondral bone fraction (BV/TV), Tb.Sp, and SBP.Th at eight weeks following sham or DMM surgery. n = 8 mice per group. (**D**) OARSI scores at eight weeks following surgery. n = 8 mice per group. (**E**) ELISA analysis of PGE2 concentrations in the subchondral bone marrow of mice following surgery. n = 5 mice per group. (**F**,**G**) Representative images (**F**) and quantitative analysis (**G**) of immunofluorescent staining of CGRP (calcitonin gene-related peptide, green) + sensory nerves in subchondral bone four weeks following surgery. Scale bar: 50 μm. (**H**,**I**) Representative images (**H**) and quantitative analysis (**I**) of immunochemical staining of osterix+ (brown) osteoprogenitors in subchondral bone four weeks following surgery. Scale bar: 50 μm. (**J**) Measurement of the 50% pressure withdrawal threshold following surgery; n = 8 mice per group. (**K**) Measurement of the pressure threshold following surgery; n = 8 mice per group. (**L**–**N**) Parameters of voluntary wheel running following sham or DMM surgery, as well as distance travelled (**L**), mean speed (**M**) and active time (**N**); n = 8 per group. * *p* < 0.05, ** *p* < 0.01, *** *p* < 0.001, and N.S. is non-significant.

To confirm that the mechanism that PGE2 affects subchondral bone remodeling is dependent mainly on sensory innervation, we generated transgenic mice where EP4 was specifically knocked out from sensory neurons (EP4^−/−^ mice). The EP4 showed an obvious increase in the whole DMM joint (Figure S2). It should be noted that the sensory nerve was absent from the articular cartilage. The microarchitectural alteration of subchondral bone in EP4^−/−^ and EP4^+/+^ mice following DMM or sham surgery by μCT was first examined. Tibial subchondral BV/TV, SBP.Th, and Tb.Sp almost returned to the levels of the controls in the EP4^−/−^ DMM mice and celecoxib-treated EP4^+/+^ DMM mice, but not in the vehicle-treated EP4^+/+^ DMM mice (Figure 6A,C). Consistent with the OARSI scores, proteoglycan loss of articular cartilage in the vehicle-treated EP4^+/+^ DMM mice was found to be significantly higher than in other groups, but the cartilage phenotype was similar in EP4^−/−^ DMM mice and celecoxib-treated EP4^+/+^ DMM mice (Figure 6B,D).

The PGE2 levels in subchondral bone were further determined. ELISA analysis found that celecoxib significantly reduced the PGE2 levels in the subchondral bone of both groups, but PGE2 levels remained higher and comparable in vehicle-treated EP4^+/+^ and EP4^−/−^ DMM mice (Figure 6B,E). In addition, the COX2 expression significantly increased in the subchondral bone in all DMM groups (Figure S6A,B). The increased sensory nerve was subsequently detected in the subchondral bone of DMM mice, and it was 20% higher in vehicle-treated EP4^+/+^ DMM mice than in the other DMM groups (Figure 6F,G). The osterix+ osteoblastic progenitors in subchondral bone marrow significantly increased in vehicle-treated DMM EP4^+/+^ mice and were primarily inhibited in celecoxib-treated mice and EP4^−/−^ mice without significant differences (Figure 6H,I). 

The pain-related phenotype was further evaluated using a behavior test. EP4^−/−^ mice and celecoxib-treated mice showed an improvement in mechanical hyperalgesia, indicated by 50% pressure withdrawal threshold and pressure threshold (Figure 6J,K), compared to the vehicle-treated EP4^+/+^ DMM mice. The vehicle-treated EP4^+/+^ DMM mice exhibited a loss of spontaneous activities, while the EP4^−/−^ mice and celecoxib-treated mice showed an improvement (Figure 6L–N). These results suggest that PGE2 in subchondral bone induces altered subchondral bone architecture and formation, as well as pain that is dependent on sensory nerves.

## 4. Discussion

PGE2 is the principal inflammation mediator for various diseases, including rheumatoid arthritis and osteoarthritis [35]. It is also regarded as one of the local regulators of bone metabolism [36]. PGE2 has been consistently reported to increase in subchondral bone in osteoarthritis, exerting osteoarthritis pain [9,26]. Recent literature has indicated that PGE2 mediates the sensory regulation of bone homeostasis and promotes the osteoblastic differentiation of MSCs [22,23]. Elevated PGE2 concentrations may result in a cascade of uneven subchondral bone formation, causing osteoarthritis development. The inhibition of PGE2 production with COX-2 inhibitors effectively attenuates the abnormal alteration of subchondral bone architecture, osteoarthritis pain, and articular cartilage degeneration. Increased PGE2 in subchondral bone activates the local sensory nerves to facilitate the further promotion of aberrant osteogenesis and pain signals via its EP4 receptor. Increased sensory innervation in subchondral bone in osteoarthritis amplifies the aforementioned process. Human osteoarthritis seems to be a more complex disease with multiple phases relating to the DMM model. It was found that the non-lesion area, moderate area, or lesion area of degenerative articular cartilage (Table 1) existed in the specimens of late-stage osteoarthritis patients who had undergone total knee arthroplasty. PGE2 concentrations were found to be positively related to the severity of subchondral bone alteration and articular cartilage degeneration in comparison to the normal controls. Therefore, the inhibition of PGE2 production or blockade of PGE2 signaling appears to be effective for the attenuation of osteoarthritis by maintaining subchondral bone architecture and the subsequent protection of articular cartilage and through pain relief. 

The causal role of subchondral bone PGE2 in osteoarthritis progression has recently been investigated [29,30,31]. Elevated PGE2 in subchondral bone results in both spontaneous osteoarthritis and rheumatoid arthritis, which is mediated by a deterioration of the subchondral bone structure [29]. Spontaneous osteoarthritis can generally be attributed to aberrant subchondral bone formation, but the exact mechanism involving elevated PGE2 remains unclear. Jiang et al. [30] explored the role of subchondral bone PGE2 in osteoarthritis, finding that osteoclasts facilitate the promotion of angiogenesis and sensory innervation with increased PGE2/EP4 signaling, which subsequently results in osteoarthritis progression and pain. Altered sensory innervation affects subchondral bone homeostasis through the induction of uncoupled subchondral bone remodeling [25]. This study found that PGE2/EP4 signaling promotes pathological subchondral bone formation via a sensory nerve. In subchondral bone, PDGF-BB derived from osteoclasts maintains the type H vessel, which might provide an environment that is beneficial for osteogenesis [30]. A previous study has reported that neither COX-2 inhibitors nor the genetic deficiency of Ptgs1^−/−^ mice or Ptgs2^−/−^ mice exhibit a chondroprotective effect in surgery-induced osteoarthritis mice [31]. The difference in results could be attributed to different methods that were used to establish osteoarthritis. In this study, the medial collateral ligament was transected, but the medial meniscus was retained in the joint. The removal of the medial meniscus results in direct contact between the tibia and the femur, and the progression of osteoarthritis is significantly aggressive. Therefore, only inhibiting PGE2 may be ineffective for the attenuation of OA.

Most PGE2 is released from osteoblasts in instances of skeletal injury, particularly adjacent to the injury site [36] and in osteoarthritic subchondral bone [26]. A previous study has shown that PGE2 in the porous endplate is derived from the various local cells, including macrophages, osteoblasts, and osteoclasts [37]. In addition, the activation of EP4 of osteoblasts by PGE2 promotes osteoblast differentiation while upregulating COX-2 expression, before further promoting PGE2 production and subsequent cascades [38]. PGE2 levels continuously increase in progressive osteoarthritic subchondral bone, together with increased osteogenesis and altered subchondral bone architecture, which is consistent with previous research [9,26,28]. Subchondral bone and articular cartilage serve as a functional unit in the joint [15]. Starting with early-stage osteoarthritis, the altered mechanical loading on subchondral bone or acute injury results in increased PGE2 production, which triggers the cascade of PGE2 production and osteogenesis and forms a vicious cycle. This induces a progressive microstructural alteration to subchondral bone and articular cartilage degeneration. 

PGE2 directly affects both subchondral bone and articular cartilage. Clinical trials have reached inconsistent conclusions regarding the effects COX-2 inhibitors have on joint function and quality of life that is improved or unchanged following the treatment of osteoarthritis, despite pain relief being improved [39,40,41,42,43]. PGE2 exhibits a protective effect on articular cartilage by binding the EP2 of chondrocytes, thereby promoting the growth of articular cartilage cells and further suppressing the expression of osteopontin [36,44]. PGE2 also inhibits proteoglycan synthesis while stimulating matrix degradation in osteoarthritis chondrocytes via the EP4 receptor, especially increasing EP4 expression in chondrocytes after osteoarthritis [45]. Increased PGE2 may exert EP4-induced cartilage degeneration, while the excessive inhibition of PGE2 levels affects the EP2-induced cartilage regeneration. In this study, proper inhibition of PGE2 production appeared to sufficiently abrogate subchondral bone hypertrophy, cartilage degeneration, and osteoarthritis pain. This is supported by clinical studies that have found that the intra-articular injection of COX-2 inhibitors provides chondroprotective effects in vivo [46].

PGE2 facilitates the promotion of osteoblastic differentiation through the sensory regulation of bone homeostasis [22,23]. Increased sensory innervation in subchondral bone was observed in this and previous studies [9,26,47]. When compared to a COX-2 inhibitor, sensory denervation by capsaicin effectively attenuated the structural alteration of subchondral bone and osteoarthritis development, despite subchondral bone having higher PGE2 levels. This suggests that the PGE2 effect is primarily abrogated by sensory denervation. PGE2 is also an essential and traditional osteoarthritis pain stimulator. Previous research has found that subchondral bone osteoclasts induce sensory innervation by netrin-1 expression, and the attenuation of aberrant subchondral bone remodeling levels reduces sensory innervation [9,47]. Through the activation of the EP4 receptor, PGE2 can regulate Na_V_1.8 neuronal modification and induce joint hypersensitivity [26,37]. PGE2/EP4 pathway-induced activation of the transient receptor potential cation channel subfamily V member 1 (TRPV1) may be another subchondral bone homeostasis and osteoarthritis pain mechanism. A previous study has shown that the PGE2/EP4 pathway leads to the activation of the TRPV1 channel, further inducing spinal hypersensitivity and spinal pain in the DRG neurons of lumbar spine instability mice [48]. In addition, the activation of the TRPV1 channel, induced by zoledronic acid, promotes osteoblast mineralization [49]. This may be downstream of the PGE2/EP4 pathway in subchondral bone osteoblasts and is responsible for increased subchondral bone volume. Application of ketamine for anesthesia could alter the effect of capsaicin and PGE2/EP4 signaling, because of the ketamine-induced potentiation of TRPV1 receptor sensitivity to capsaicin [50]. However, the terminal elimination half-life of ketamine is approximately 1.26 h [51]. Therefore, a single dose of ketamine with a dose of 80 mg/kg will not affect this conclusion. Despite the distribution of EP4, the lack of a TRPV1 channel in osteoclasts limits the effect PGE2 has on osteoclasts [49]. 

Despite good outcomes for patients with moderate arthritis [39,40], some adverse events increased following the application of COX-2 inhibitors in osteoarthritis patients, including abdominal pain, hypertension, heart failure, and edema [52]. The systemic application of COX-2 inhibitors can effectively reduce the PGE2 level in subchondral bone while also significantly limiting PGE2 production in other tissue. The EP4 receptor is widely distributed in both cartilage and subchondral bone, including osteoblasts and osteoclasts in both mice and humans [30,45,53,54]. In addition, there is a significantly increased expression of EP4 receptors in osteoarthritic subchondral bone and articular cartilage [30,45,53,54]. Previous research has found that EP4 receptor antagonists can effectively inhibit PGE2/EP4 signaling in animals [30,55,56], specifically inhibiting the binding of PGE2 and EP4 receptors. Therefore, a specific blockade of the EP4 receptor represents a future strategy for the disease modification of OA by inhibiting the PGE2/EP4 pathway. The acceleration of PGE2 inactivation could also be an alternative to osteoarthritis treatment. Nicotinamide adenine dinucleotide (NAD+)-dependent 15-hydroxyprostaglandin dehydrogenase (15-PGDH) is a key enzyme that is involved in the catabolism of prostaglandins, which leads to the metabolic inactivation of excess PGE2 [57]. The local application of histone deacetylase inhibitors and peroxisome proliferator-activated receptor-gamma agonists, which can induce the expression of 15-PGDH, may be capable of avoiding the side effect of decreased production of PGE2 caused by COX-2 inhibitor. 

PGE2 interacts with other signaling pathways or factors to facilitate the induction of aberrant subchondral bone architecture, including IGF-1, PTH, TGF-β, BMP-2, and abnormal angiogenesis [10,36,58,59,60]. Increased serum IGF-1 plays a critical role in higher osteoarthritis incidence [61]. PGE2 has a strong effect on IGF-1 gene promoter activity, and increased IGF-1 intensively results in the abnormal formation and altered architecture of subchondral bone [62,63]. Increased IGF-1 and PGE2 proteins in osteoarthritic subchondral bone also downregulate the PTH receptor expression of osteoblasts. PTH resistance impairs PTH-dependent cyclic adenosine monophosphate (cAMP) signaling in osteoblasts, causing subchondral bone sclerosis to be induced [64]. Overactive TGF-β in subchondral bone results in the formation of osteoid islets and increases angiogenesis by causing MSC clusters to form [10]. TGF-β also intensively increases COX-2 expression and PGE2 production [65]. PGE2 production may also indirectly increase active TGF-β levels by inducing an altered architecture of subchondral bone. Skeletal injury promotes the local production and release of BMP-2 and PGE2 [36]. Both BMP-2 and PGE2 act via the MAPK signaling pathway by activating their specific receptors on osteoblasts, which ultimately leads to the expression of alkaline phosphatase, osteopontin, and osteocalcin, and subsequent subchondral bone formation and sclerosis [36]. 

## Figures and Tables

**Table 1:** Antibody information.

Reagent Type	Designation	Source	Identifiers
primary antibody	calcitonin gene-related peptide (CGRP)	ab81887	Abcam
	MMP13	ab39012	Abcam
	osterix	ab22552	Abcam
	COLX	ab58632	Abcam
	osteocalcin	M188	Takara
	COX-2	ab15191	Abcam
secondary antibody	goat anti-rat IgG (Alexa Fluor® 594)	ab150160	Abcam
	goat anti-mouse IgG (Alexa Fluor® 488)	ab150113	Abcam
	goat anti-rabbit IgG (Alexa Fluor® 488)	ab150077	Abcam
	goat anti-rabbit IgG (HRP)	ab205718	Abcam

**Table 2:** Demographic information of patients.

	Normal	Osteoarthritis
Sample size	8	8
Gender (M/F)	4/4	4/4
Height (cm)	174.3 ± 4.1	170.1 ± 3.9
Body weight (kg)	67.8 ± 5.9	72.1 ± 4.9
Age (y)	36.5 ± 4.7	62.3 + 2.5
BMI	23.6 ± 2.6	25.2 ± 3.1
Varus deformity (°)	N/A	11.4 ± 2.6
Knee society score [34]	N/A	54.2 ± 5.7

**Table 3:** Articular cartilage degeneration grade.

Classification	Detail
Non-lesion area	intact cartilage
Moderate area	minimal or overt fibrillation of cartilage
Lesion area	partial cartilage erosion, and exposures of subchondral bone

## Data Availability

Not applicable.

## Supplementary Materials

The following supporting information can be downloaded at: https://www.mdpi.com/article/10.3390/cells11172760/s1, Figure S1: PGE2 level is positively related to osteoarthritis pain in the DMM model; Figure S2: Overviews of the mouse knee stained with EP4 (upper panel) and COX2 (lower panel); Figure S3: COX-2 inhibitor exhibits dose-dependent effects on articular cartilage and subchondral bone; Figure S4: Systemic celecoxib effectively inhibits PGE2 production in knee joints; Figure S5: Sensory denervation reduces pain and preserve activity; Figure S6: COX-2 exhibits increase expression in the DMM subchondral bone; Table S1: Mouse information.
