# Supplementary material for: Inhibition of PGE2 in Subchondral Bone Attenuates Osteoarthritis
Source: Cells. 2022 Sep 5;11(17):2760. doi: 10.3390/cells11172760 (PMC9454853; doi:10.3390/cells11172760)
Supplement: Supplementary file 1 [file cells-11-02760-s001.zip › cells-1859098-supplementary.pdf]

# Inhibition of PGE2 in Subchondral Bone Attenuates Osteoarthritis

Supplementary Materials

Table S1. Mouse information.

| | | SOFG and CT | Other histology | PGE2 analysis | Double labeling |
|---|---|---|---|---|---|
| Fig 1 | number* | 80, n=8 | | 50, n=5 | / |
| | harvest time | 0, 1, 2, 4, 8 weeks after surgery | | | / |
| Figs 3 and 4 | number* | 24, n=8 | 24, n=8 | / | 15, n=5 |
| | harvest time | 8 weeks after surgery | 4 weeks after surgery | / | 4 weeks after surgery |
| Fig 5 | number* | 24, n=8 | 24, n=8 | 24, n=8 | / |
| | harvest time | 8 weeks after surgery | 4 weeks after surgery | 4 weeks after surgery | / |
| Fig 5 | number* | 48, n=8 | 48, n=8 | / | / |
| | harvest time | 8 weeks after surgery | 8 weeks after surgery | / | / |
| Fig S2 | number* | 30, n=6 | / | / | / |
| | harvest time | 8 weeks after surgery | / | / | / |
| Fig S3 | number* | / | 15, n=5 | / | / |
| | harvest time | / | 4 weeks after surgery | / | / |

number* is shown in format of total number and number per group.

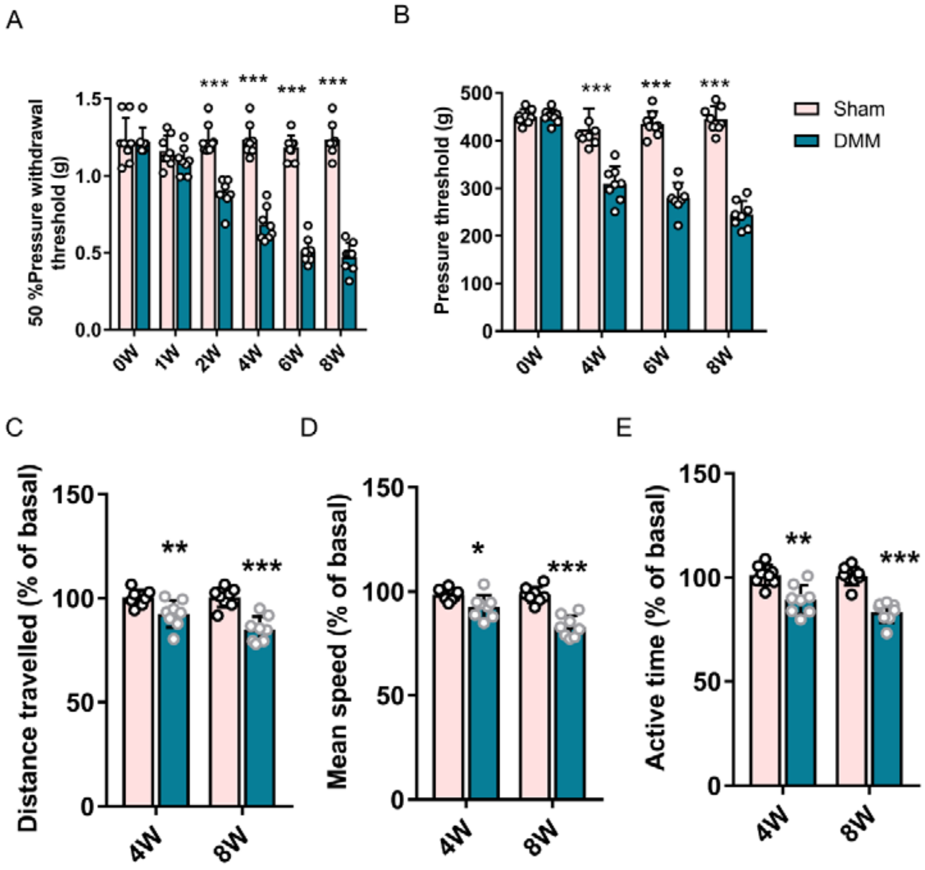

**Figure S1. PGE2 level is positively related to osteoarthritis pain in the DMM model. (A)** Measurement of the 50% pressure withdrawal threshold of mice after DMM or sham surgery; n = 8 mice per group. **(B)** Measurement of the pressure threshold of mice after DMM or sham surgery; n = 8 mice per group. **(C-E)** Parameters of voluntary wheel running of mice after DMM or sham surgery including active time **(C)**, mean speed **(D)**, and distance traveled **(E)**; n = 8 mice per group. * $p<0.05$, ** $p<0.01$, *** $p<0.001$, and N.S. means non-significant.

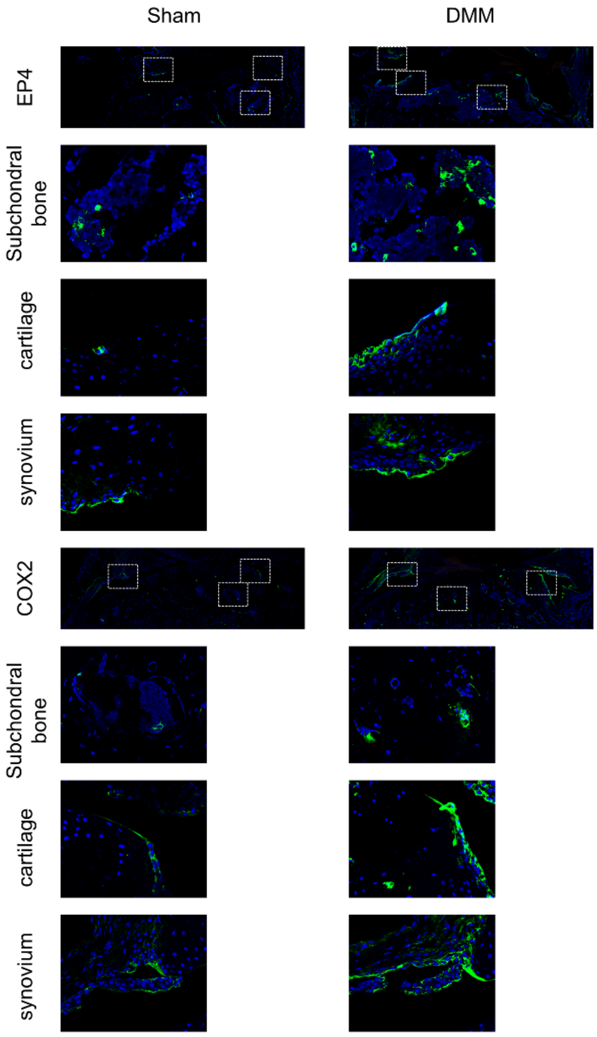


**Figure S2.** Overviews of the mouse knee stained with EP4 (upper panel) and COX2 (lower panel). Left, an overview of sham group mice knee section stained. Right, an overview of DMM group mice knee section stained.

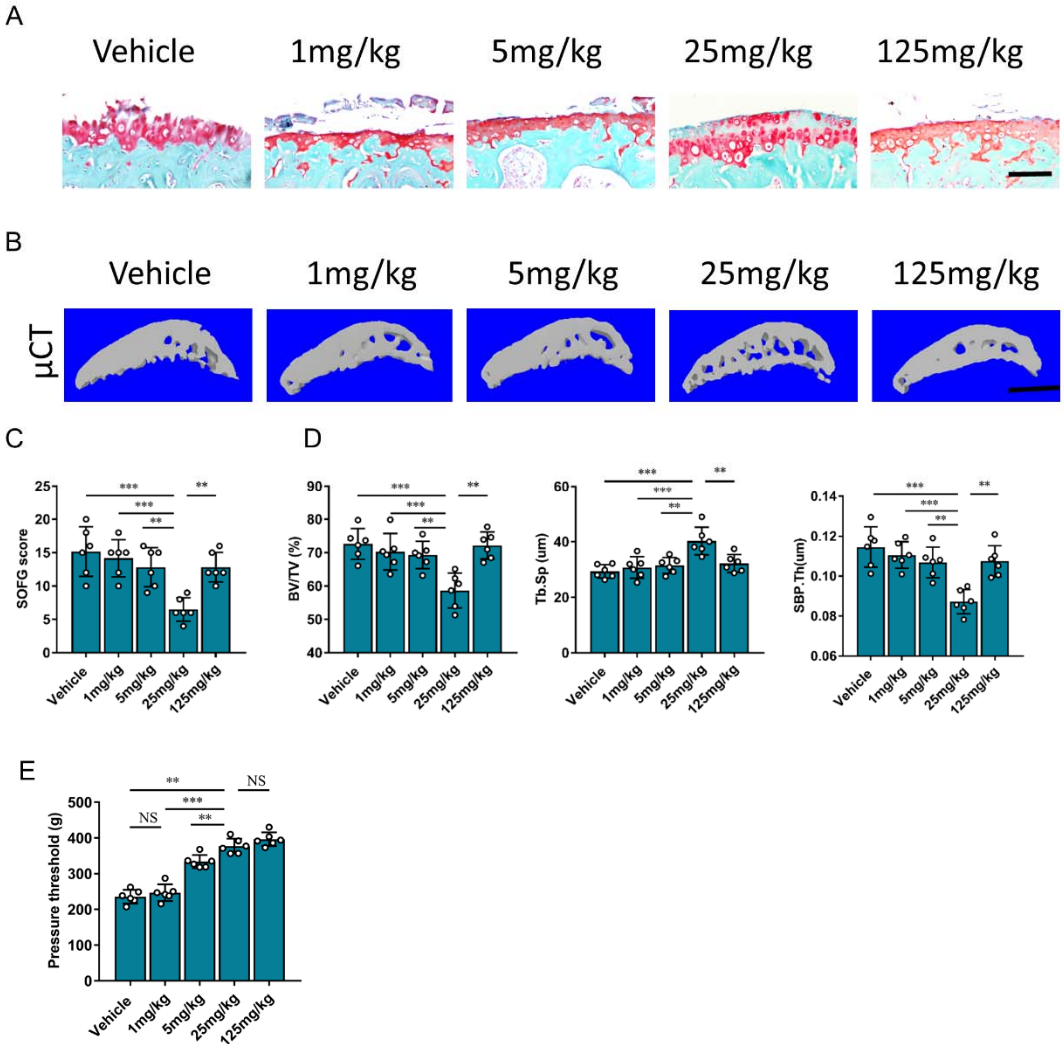


**Figure S3. COX-2 inhibitor exhibits dose-dependent effects on articular cartilage and subchondral bone. (A)** SOFG staining of the medial area of tibial subchondral bone (sagittal view). Scale bar: 100 µm. **(B)** Three-dimensional microcomputed tomography (µCT) images of tibial subchondral bone (sagittal view). Scale bar: 500 µm. **(C)** OARSI scores. n = 5 mice per group. **(D)** Quantitative analysis of subchondral bone fraction (BV/TV), Tb.Sp, and SBP.Th. n = 5 mice per group. **(E)** Measurement of the pressure threshold of mice. n = 6 mice per group. * p<0.05, ** p<0.01, *** p<0.001, and N.S. means non-significant.

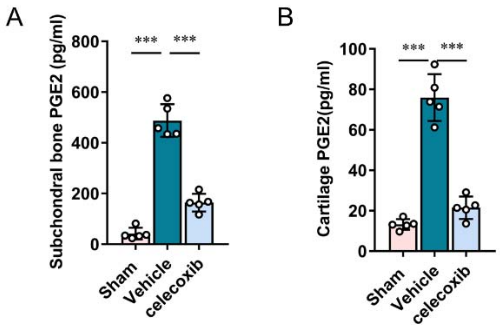


**Figure S4. Systemic celecoxib effectively inhibits PGE2 production in knee joints. (A)** ELISA quantitative analysis of PGE2 levels in mouse subchondral bone 4 weeks after DMM or sham surgery. n = 5 mice per group. **(B)** ELISA analysis of PGE2 protein levels in the articular cartilage four weeks after DMM or sham surgery. n = 5 mice per group. * p<0.05, ** p<0.01, *** p<0.001, and N.S. means non-significant.

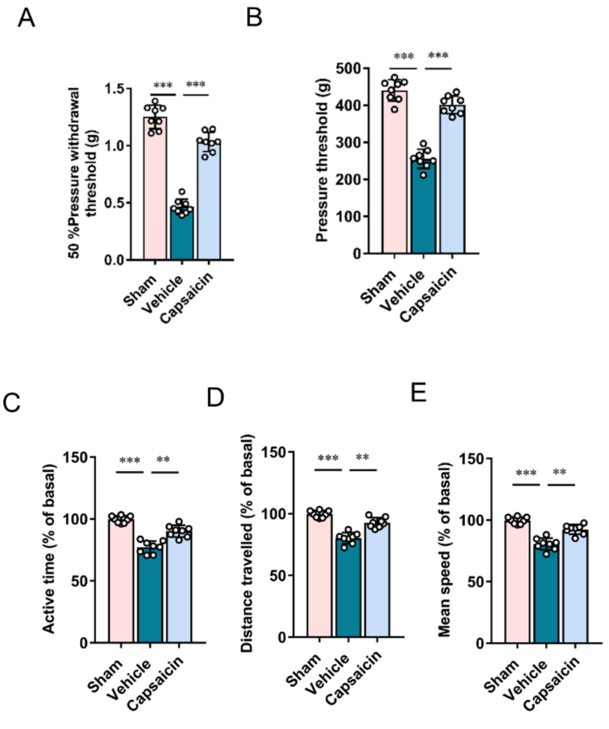


**Figure S5. Sensory denervation reduces pain and preserve activity. (A)** Measurement of 50% pressure withdrawal threshold after surgery; n = 8 mice per group. **(B)** Measurement of pressure threshold after surgery; n = 8 mice per group. **(C-E)** Parameters of voluntary wheel running after surgery, active **(C)**, mean speed **(D)**, and distance traveled **(E)**; n = 8 mice per group. * p<0.05, ** p<0.01, *** p<0.001, and N.S. means non-significant.

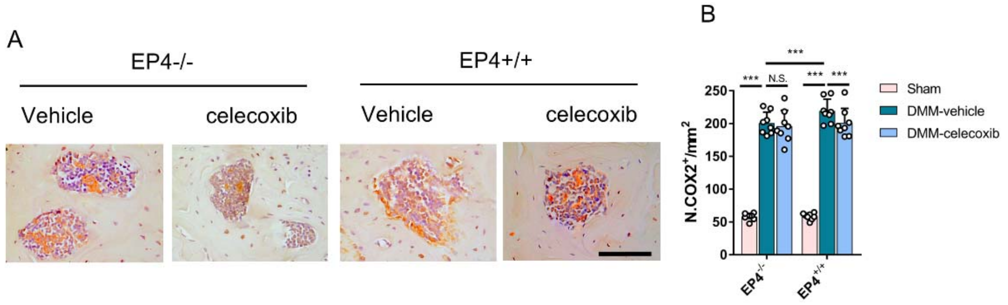


**Figure S6. COX-2 exhibits increases expression in the DMM subchondral bone.** Representative images **(A)** and quantitative analysis (B) of immunochemical staining of COX2+ (brown) osteoprogenitors in subchondral bone four weeks following surgery. Scale bar: 50 μm. n = 8 mice per group. * p<0.05, ** p<0.01, *** p<0.001, and N.S. means non-significant.
